# Identification of Animals

**Published:** 1891-11

**Authors:** R. S. Huidekoper

**Affiliations:** Veterinarian


### IDENTIFICATION OF ANIMALS.*

By R. S. Huidekoper, M.D., Veterinarian.

There is perhaps no part of veterinary practice or animal commerce, in which there is a greater want of method, laxness in detail, and carelessness in recording, than in the description of the subjects handled, and in the noting of points, which will establish the future identification of an animal. For the ordinary layman an equine is a horse or a mule, a bovine a cow, and a canine a dog, irrespective of age, color, sex, or condition of servitude. A stallion is a rarity, a steer is a beef animal, and by many a bitch, bull, or gelding, is not considered as mentionable in polite society. This inattention to definite terms leads not only to vagueness in veterinary writings, but also frequently causes the writer to commit errors in English, which could have been avoided, by a definite fixation of the subject matter.

In the description of an animal for veterinary purposes, whether in an expert examination for soundness, in judging, or in a legal controversy, or in a clinical description, or for registration in a stud-book, a definite course of examination and a methodical system of recording should be observed. If this is done, and the written description follows the same form in every case, it educates the observer to note the small, and sometimes apparently trivial differences, which at times are the important points upon which an identification is based.

The table below outlines the course to be followed in an ordinary record of the identification of an animal:

I. Description of Animal. *a*, Species of animal, race, family; *b*, Sex; *c*, Age; *d*, Height and weight; *e*, Color; *f*, Accidental markings.

II. Description of Abnormal or Pathological Alterations.

With the domesticated animal custom has established certain specific terms which define species, sex and age, and these should be used according to their exact meaning, and with no other meaning so far as possible, in all expert writings.

Genus Equus; *Species caballus*. Specific term, Horse. The animal is—a *Foal*, irrespective of sex, from birth until weaned; a *Weanling*, when weaned until it becomes a *Yearling*. The male

* Read before the United States Veterinary Medical Association, at Washington, Sept. 16th.

animal is—a *Colt*, until the mouth is made, or until castrated; custom has, however, accepted the first indication of the corner teeth or four years, as the age at which he becomes a horse ; a *Gelding*, after castration, at any age; a *Horse* or *Stallion* after the the mouth is made, or earlier, if he stands for service; a *Ridgling*, if one testicle has not descended to the scrotum. The female is—a *Filly*, until the mouth is made or, until bred; a *Mare*, after the mouth is made, or sooner if bred.

*Species asinus.* Specific term, Ass. The ass is—a *Foal*, until weaned; after that the male animal is a *Jack*; the female animal is a *Jenny*. The male mule is known as a *Jack Mule*, irrespective of gelding, and the female as a *Jenny Mule*. The hybrid foal of the male ass and the mare is the true mule. That between the stallion and the female ass is called the *Hinny*.

GENUS BOS ; *Species domesticus.* Specific term, Neat Cattle. The animal is— a *Calf* until six months old (the natural time for weaning); a *Bullock* is a young bull, or any male of the ox kind ; a *Bull* is the male animal; a "*Steer* is the castrated male of neat cattle. He is called an ox calf, or bull calf, until he is twelve months old, a steer until he is four years old, and after that an ox or bullock," *Youatt;* an *Ox* (*vide*) "steer"; a *Stag* is a castrated male; a *Heifer* is the female until bred, or until the mouth is made; a *Cow* is the female after breeding, or when the mouth is made.

GENUS OVIS ; *Species aries* Specific term, Sheep. The animal is—a *Lamb* until a year old; a *Ram* or a *Tup*, when male over eighteen months old, and has its first intermediate permanent teeth; a *Ewe* when female over eighteen months old, and has its first intermediate teeth; a *Wether*, when a castrated male; a *Hog-Hogget* is the young sheep before it has been shorn.

GENUS CAPRA ; *Species hircus.* Specific term. Goat. The animal is—a *Kid*, until a year old; a *Billy* is the male; a *Nanny* is the female.

GENUS SUS; *Species scrofa.* Specific term, Swine, Pigs, Hogs. The animal is—a *Suckling* until weaned; a *Roaster*, from four until eight weeks old; a *Pig* until a year old, male or female; a *Porker*, *Porket*, or *Porkling* is a young hog or pig; a *Boar* is the adult male; a *Sow*, the adult female; a *Shoat*, *Shote* or *Shoot* is a growing hog; a *Barrow* is a castrated hog; a *Farrow* is a litter of pigs.

GENUS CANIS ; *Species domesticus.* Specific term, Dog. A *Puppy* is—the young; a *Dog* is the male; a *Bitch* or *Slut*, the female, (the former term is preferable).

GENUS GALLUS ; *Species domesticus.* Specific term, Chickens, Barnyard Fowls, Pullail. A *Cock* is the male ; a *Cockerel* is a young cock; a *Stag* is a young game cock; a *Capon* is a castrated male; a *Hen* is the female; a *Pullet* is the young female; *Poultry* are the fowls fed for the table.

AGE. Beyond the definitions made by the specific terms just mentioned, the question of age is too long a subject to enter into in a paper of this kind, and is also one which has been made the subject of such minute study and description in numerous writings, that they can be referred to, for accuracy.

HEIGHT. The horse varies from 9 hands to 22 hands in height; under 14 hands he is known as a pony; cobs measure from 14 to 15½ hands. Some of the great Belgian and English draft horses reach 18 hands or even more. Barnum showed, a few years ago, an ungainly broken-kneed bay coach-bred gelding, that measured 22 hands. On a written certificate the heights should be given as under standard (S) or estimated (E). The height is taken at the highest bony point of the withers, the spinous process of the seventh dorsal vertebra. Care should be taken to see that the horse being measured is standing on an exact level with the examiner, and with the instrument used. The ordinary form of instrument used is the *standard*, a rod six feet in height with a movable cross bar, the latter usually fitted with a spirit level. Care should be taken to see that the upright is perfectly vertical, as a small inclination will make an important difference in the horizontal bar. When there is a decided difference in the height of the withers and croup, as sometimes occurs, it should be noted, but the record is taken from the former. It must be remembered that in double teams, the form and style in carrying the head will frequently render two horses a good match, when the standard shows a decided difference in their height.

WEIGHT. The question of weight, in the description of horses, is a custom almost exclusively American. The average weight of an ordinary horse is about 1000 lbs.

| | |
|---|---|
| Ponies are under, - - - - - | 800 lbs. |
| Light roadsters, - - - - - - | 950 " |
| Ordinary roadsters and saddle horses, - | 950 " to 1150 lbs. |
| Coach horses, - - - - - - | 1000 " " 1350 " |
| Light draft horses, - - - - - | 1000 " " 1350 " |
| Medium draft horses, - - - - | 1350 " " 1500 " |
| Heavy draft horses, - - - - | 1500 " and over. |

With a severe fever or other illness a horse may loose 25 lbs. to 40 lbs. a day, 200 lbs. in a week.

COLOR. The "coat" of animals is made by an ensemblage of the color of the skin itself (epidermic pigment), the character and color of the hairs, and the products of secretion and excretion of the glands of the skin (sebaceous and sudoriparous glands).

In wild animals the coat is almost invariably distinctive of the species of animal, although varying frequently, somewhat with the age of the animal, and with the sex. In all animals the hairs are generally coarser and longer over the neck and withers in the male than in the female; and the color of the former is, in most species, darker and more pronounced than in the latter. But while wild animals have usually a uniform color, domesticated animals, and many wild ones, which have been bred in captivity, are subject to great variation in color, and their coats are subject to the introduction of white, which is rare in wild animals.

THE SKIN. The skin may be pigmented throughout with a deposit of a leaden or brick dust color, which may even extend to the mucous membrane of the nostrils, tongue and hard palate, or the pigment may be entirely absent, or may be absent only from certain parts; in the latter case, it is usually absent from the neighborhood of the natural openings, the lips, the eyelids, the genitals and the anus, and from the extremities, pasterns, fetlocks, legs, and face.

THE HAIRS. *Character.* The hairs are of two sorts; the hairs proper, which cover the surface of the body, and are short, fine and soft, and lie closely together in a definite direction, for each given part of the body. These are shed annually or oftener. The longer coarser hairs, which, under the name of mane, forelock tail, tentacles, eyelashes, moustaches, and whiskers, grow from the crest of the neck, the tail, the eyelids and the lips, and on the fetlocks of coarse bred animals, are persistant. The hairs of the hog are always coarse and are called bristles, and those of the sheep, of great fineness and crinkled, are known as wool; both of these terms are used for the hair of the other animals, when, in its texture, it resembles them.

*Color.* The colors of animals are not as brilliant as those of a flower or of the plumage of birds, but we can recognize that, like these, they are based upon the primitive colors of the prism, red, blue, and yellow. In the horse we have as primitive colors a modification of—*Red*, bay and chestnut; *Blue*, steel-grays and mouse color; and, *Yellow*, duns; *White*, absence of all colors, and, *Black*, presence of all colors. From modifications in the shades and tints of these, and from combinations of them we have the colors of the coats. Professor Neuman divides the coats into three categories: 1, *Primitive coats;* 2, *Derived coats;* and 3, *Conjoined coats*.

*Primitive coats*, are those which the animal has at birth. *Derived coats*, are those which appear after birth, and are due to the introduction of white. *Conjoined coats*, are those in which there is the presence in the same animal of two coats, primitive or derived.

1. *Primitive coats.* Form three groups: A. *Simple coats*, with but one single color, as black or sorrel. B. *Composite coats*, formed of hairs of two colors, black for the mane, tail and extremities, and yellowish, reddish or bluish, for the body, as the dun, bay or mouse color. C. *Mixed coats*, are made of hairs composed of two colors, usually yellow at the base and black on the end; this is a rare color, and when it exists in the horse, is often improperly confounded with the roans.

A. *Simple coats.* The simple coats are those made of hairs of but one color, the *Black*, *Sorrel* or *Chestnut;* Goubaux and Barrier exclude the *White* as a color, as in the so-called white horse there is invariably more or less trace of black or other color making it a very light gray. If it can be shown that absolutely white (not albino) horses exist, then, white can be included as a simple coat.

a. *Black.* The black may be a dull, uniform, dead black, without any reflecting tints, or the latter may be so marked in reddish or yellowish hues as to confound the coat with that of a brown bay. It may have the brilliant reflection of jet.

b. *Chestnut* or *Sorrel.** This is a yellowish or brownish red, which comprises many shades. Commencing with the lightest in colors there is: 1. The *Cream color*, which may be subdivided, as light, ordinary or dark; 2. The *Light sorrel*, has a decidedly yellowish tint, like the coat of the lion. An ordinary chestnut turned to pasture for a month, will sunburn into this color; 3, *Sorrel* or *chestnut* (ordinary) is a cinnamon color, the hairs of mane, tail and legs, must have the same color as those of the body; 4, *Washed sorrel*, is a degenerate light sorrel, but the legs, mane and tail are very light or almost white; 5, *Dark sorrel*, is a brownish cinnamon color; 6, *Cherry sorrel*, has a decided bright red tint; 7, *Chestnut* (proper) is a rich uniform brownish red, like that of a ripe

* These terms are used as synonomous.

chestnut; 8, *Burnt sorrel*, is a dark rich brown, like roasted coffee. Horses of this color invariably have more or less white hairs sprinkled over the body, and a mane and tail of almost a white or silver color. According to the reflection of the tints of a sorrel, it may be golden, fox (copper), or bronze. White marks on the head and legs of sorrels are common.

B. *Composite coats.* Composite coats are those with two distinct colors, a reddish, yellowish or bluish one for the body, and black for the mane, tail and legs; they are the *dun*, *bay* and *mouse* color.

a. *Dun.* The dun has yellowish hairs over the body, to the knee and hock and over more or less of the face. The mane, tail, and hairs on the cannon and fetlocks are black. A dun may be *light*, *ordinary* or *dark*, in the latter case taking the name of *Buckskin*. Duns frequently have a black line running along the centre of the back, the black cross of the ass on the withers, and the transverse black marking of the Zebra on the legs. The light dun is sometimes almost the same as a *cream color*, but can always be distinguished from it by the presence of black on the extremities. The dun is known in French as an *Isabelle;* according to Bouillet, *Dictionnaire d'Histoire et de Géographie*, the Archduchess Isabelle of Austria, daughter of Philip II of Spain and and ruler of the Netherlands, accompanied her husband at the seige of Ostend, and made a vow not to change her linen before the conquest of the place. The seige lasted from 1601 to 1604, and the color of the chemise of the Princess gave origin to the name.

b. *Bay.* In the bay the color of the body is a red, which may vary from a cherry to a mahogony, and is distinguished from any similar shade of sorrel, by the presence of the black mane, tail, and legs, although in the last the black may be reduced to a narrow circle of black hairs around the coronary band. The *Bays* are: 1. *Light Bay*, these frequently have the black cross and zebra stripes on the withers and fore legs; 2. *Bay* (ordinary) distinctly red; 3. *Blood Bay* (mahogony) darker; 5. *Dark Bay* approaching brown; 5. *Brown Bay* or *Brown;* becomes nearly black in Winter, but always has a reddish tint around the muzzle, armpits, belly, flanks and thighs, while the sunburnt *black*, in Summer, is black in the same places.

White markings on bays are usually less frequent and less extensive than on sorrels.

c. *Mouse color.* The mouse color is less frequent in the horse than in the ass and mule. It is an ashy blue over the body, while the mane, tail and legs of the animal are black. It is frequently accompanied by the black cross and zebra stripes.

C. *Mixed coats.* This is a rare color, composed of hairs which are yellow at the base and dark at the tips, like those of wolves, and some wild animals. It is probably usually confused with the roans, but should not be.

2. *Derived Coats.* Derived coats are those due to the introduction of white into the primitive coat at some period after birth; they are the *Grays* (including white) and *Roans.*

A. *Gray.* Goubaux and Barrier say : "Classically, gray "is a mixture of white and black hairs. Practically, it is far from "being so. Take by chance ten gray horses, and it is readily "seen that this definition is not sufficient. The dark hairs *are* "*not always black.* They are often brown, chestnut or reddish "(bay), more rarely yellow. The *White* hairs are sometimes "washed yellow." Brivet says: "The gray coat is excessively "variable in tint; it is a sort of chaos, mixed with hairs of "different shades."

The gray is a mixture of light and dark hairs over, not only the body, but also in the mane, tail and legs; it can have for basement color, black, bay, sorrel or dun. A gray may be: 1. *Very little gray*, showing only a few dark hairs, usually called white; 2. *Light gray*, with more dark hairs, especially on mane, tail and legs; 3. *Gray* (ordinary), with about an equal mixture of white and black hairs; 4. *Dark gray*, with a predominence of dark hairs; 4. *Iron gray* (steel), with a bluish tinge, like that of a freshly broken piece of steel; (a darker shade might be called *slate colored;*) 6. Grays can further be distinctly characterized as *Dirty*, *Dun* or *Roan* grays.

Grays usually become lighter as the animals become older, so that at different ages the same animal would need an altered description. The *White* if recognized as a coat, or in the ordinary case, when it is a coat derived with age, from the grays, is *milk*, *china* (bluish), *dirty* or *rose* white; these qualifications indicate the tint given to the white, without further explanation.

The *Strawberry roan* (or Peach) is a sorrel, with numerous white hairs in body, mane and tail; it may be light or dark. A strawberry roan is always much lighter after clipping.

B. *Roan.* The roan has a coat with three colors of hairs, black, reddish and white, that is a body with numerous white hairs over the body. It may be *light, medium* or *dark.* According to the color of the hairs which predominate and the distinctive tint of the roan, it may be further qualified as a *blue* roan, a *red* roan (not to be confounded with *strawberry* roan, white and chestnut), a *white* roan, etc.

3. *Conjoined Coats* are those in which the same animal has two or more distinct primitive or derived coats on different parts of the body. They are represented by the *Piebald* or *Skewbald* which has a mixture of large white patches with other colors. The piebald strictly is a mixture of white and black, like the magpie from which it takes its name, but by custom includes the others; "*pie*" refers to the white; for more definite description, we may consider: a *pied-black*, a *pied-sorrel*, a *pied-bay*, a *pied-roan* or a *pied-gray* as a black, sorrel, bay, roan or gray with larger patches of white than of the dark color, while a *black-pie*, a *sorrel-pie*, a *bay-pie*, a *roan-pie* or a *gray-pie* is one in which the white is of less extent. We may also describe the horse as being *pied*, on near or off, belly, side, withers, or wherever the white may be. The color of the mane and tail should be indicated if the *pie* is a mixed one.

In rare cases there are horses, with conjoined coats of black and bay, two colors of bay, dun and gray, etc. These may be indicated by special description as *pied* of such and such color.

*Spotted* (Tiger spots). Spotted horses are found, especially in Denmark and from the valley of the Danube, and in the United States from Virginia and Michigan, which can be described as of such or such a color, spotted with such or such a color, the size of spots and location to be given.

SPECIAL MARKINGS. In addition to the color of the coat, it may have peculiar growth of the hair, tints, or discolouration, which give it a characteristic effect, and serve to identify the animal.

They may be divided into: 1. *General Markings;* 2. *Markings of the head;* 3. *Markings of the body;* 4. *Markings of the legs;*

*General Markings* comprise reflection of color, dark hairs, white hairs, black hairs, reddish hairs, cowlicks, and discoloration of the skin.

*Reflection of color* may be called: *Jet,* if a brilliant black; *Silvery,* if a bluish, porcelain white; *Golden,* for rich sorrels, bays and duns; *Bronzed*, for metallic reds and browns; *Watered*, when presenting alternate shades or undulations of color.

*Darker hairs*: — *Dapples* are spots usually the size of a silver dollar composed of darker more brilliant colors, than those of the rest of the body.

*White hairs*: — *Absence* of white hairs defines a horse as *solid* in color; *Scattered* white hairs, when not of sufficient number to make a derived coat, should be noted; *Fringed*, is a mixture of white hairs and those of the coat of the animal surrounding a circumscribed white spot.

Careful attention should be given, to noting the difference of natural white markings and not to confound them with *Accidental white*, which is the result of wounds, accidental scars, rubbing of harness or saddle, and blisters.

*Black hairs*:—*Fly Specked* is said of small spots or black hairs, seen most frequently in grays, sometimes in chestnuts, bays and duns; *Ermined* is the presence of larger spots of black, occurring most frequently in or along the borders of white markings; *Burnt* is the black shading of various coats, most commonly seen in sorrels.

*Red hairs*:—*Flea Bitten* is said of small spots of red hairs over the body and more frequently on the head and face, this is most frequently seen in old grays; *Sun burnt* is the reddish hue often seen in blacks; *Roan* (adjective) is the qualification used when reddish hairs appear over a gray, as *roan gray*.

*Direction of hairs*: — *Cowlicks*. Cowlicks are hairs running in irregular directions from, or to, a given point; if the first they are eccentric, if the second concentric. They occur on all horses in the centre of the forehead, on the breasts, and on the flank. They may occur at other parts of the body, and should then be noted marking the size of the cowlick and direction of the hairs. They are apt to occur in rich colored coats, and are often very distinctive of family. The trotting horse *Commonsense* is peculiarly marked with them. The Arabs consider them a mark of great quality. *Feathered* is the term used when the divergence or convergence of hairs takes place from an elongated centre.

*Discoloration of the hairs or skin*:—*Washed* is the term given to the faded tint seen with many coats. In bays it is found in the light or yellow colored legs; it is frequent on the legs of sorrels. *Leprous spots* denote the absence of pigment from the skin in spots or patches of variable size. They are frequent on the genitals and lips, often occur on the eyelids, anus, and under the white hairs on the extremities, and may be found on any part of the body. If these patches have spots of pigment in them, they are termed *Marbled.* Geoffroy St. Hilaire, Curnieu and Goubaux have noted horses entirely denuded of hair.

PECULIARITIES OF THE HEAD. White may be present in variable extent, but is usually in more or less definite form and takes with each a specific name.

*A Star* is where the hairs make an eccentric cowlick, running in all directions; *A Flame* is where they run in one direction from a cowlick, and the direction should be noted as to right or left or if in the rare direction downwards; *A Shield* is in the form indicated by the name; *A Crescent* (quarter moon) faces up or down, to right or to left and should be so noted; *A Blaze* is a white stripe down the face, it may be to right or left, may commence above with a star and may terminate below with a white nose or with leprous markings; *A Snip* is a little stripe of white on the nose; *Bald Face* is where the whole face is white.

Any of these markings may be ermined or fringed. The face may be fly-specked, or flea-bitten.

*Moustaches* or excessive growth of rather coarse hairs are at times seen in the upper lip of horses, especially those with Irish and Breton blood, from Vermont and Canada, Hackneys, etc.

Grays, roans and duns have at times very dark, almost black faces, which are characteristic.

*Wall Eyed* is applied to eyes in which the dark pigment of the iris is replaced by a light gray or bluish white. It may be complete or incomplete, and may affect only one or both eyes.

Brivet says that wall eyed horses do not see well in the dark. The ordinary pigment of the eyes is sometimes replaced, in one or both, by a tawney yellow or wine color.

MARKINGS ON THE BODY:—*Mule Stripe* is a black or dark red stripe extending on the median line from the withers to the base of the tail; *The Cross* is a stripe at right angles to the Mule Stripe from the withers down the shoulder; *White or Washed Hairs* may occur in patches over the body; *Zebra Stripes* are transverse black bands, seen usually on the upper arm, sometimes as low as the knee.

MARKINGS OF THE LEGS.—White on the extremities is described as "white," "coronary," etc.; "pastern" or "fetlock" when it simply surrounds the coronary band, covers the pastern or reaches the fetlock; *A Stocking* is white reaching to the knee or hock; *A Half Stocking* reaches half way up the cannon. These white markings may be *incomplete* (internal or external), *fringed*, *ermined*, *flea bitten*, etc. When white occurs on one leg only, the leg is indicated. When on two, they are defined as anterior biped, posterior biped or diagonal biped (left or right), according to the fore leg of the diagonal. When three legs are white, they are described white *except* the odd leg.

Identification may be made more complete by indicating the complete or partial want of pigment in any of the hoofs.

